# Matrix Metalloproteinases and Glaucoma Treatment

**DOI:** 10.1089/jop.2019.0146

**Published:** 2020-05-06

**Authors:** Robert N. Weinreb, Michael R. Robinson, Mohammed Dibas, W. Daniel Stamer

**Affiliations:** ^1^ Hamilton Glaucoma Center, Shiley Eye Institute and Viterbi Family Department of Ophthalmology, University of California San Diego, La Jolla, California.; ^2^ Allergan plc, Irvine, California.; ^3^ Department of Ophthalmology, Duke University, Durham, North Carolina.

**Keywords:** bimatoprost, extracellular matrix, glaucoma, intraocular pressure, matrix metalloproteinase, prostaglandin analog

## Abstract

Matrix metalloproteinases (MMPs) are a family of proteolytic enzymes that degrade extracellular matrix (ECM) components such as collagen and have important roles in multiple biological processes, including development and tissue remodeling, both in health and disease. The activity of MMPs is influenced by the expression of MMPs and tissue inhibitors of metalloproteinase (TIMPs). In the eye, MMP-mediated ECM turnover in the juxtacanalicular region of the trabecular meshwork (TM) reduces outflow resistance in the conventional outflow pathway and helps maintain intraocular pressure (IOP) homeostasis. An imbalance in the MMP/TIMP ratio may be involved in the elevated IOP often associated with glaucoma. The prostaglandin analog/prostamide (PGA) class of topical ocular hypotensive medications used in glaucoma treatment reduces IOP by increasing outflow through both conventional and unconventional (uveoscleral) outflow pathways. Evidence from *in vivo* and *in vitro* studies using animal models and anterior segment explant and cell cultures indicates that the mechanism of IOP lowering by PGAs involves increased MMP expression in the TM and ciliary body, leading to tissue remodeling that enhances conventional and unconventional outflow. PGA effects on MMP expression are dependent on the identity and concentration of the PGA. An intracameral sustained-release PGA implant (Bimatoprost SR) in development for glaucoma treatment can reduce IOP for many months after expected intraocular drug bioavailability. We hypothesize that the higher concentrations of bimatoprost achieved in ocular outflow tissues with the implant produce greater MMP upregulation and more extensive, sustained MMP-mediated target tissue remodeling, providing an extended duration of effect.

## Introduction

Matrix metalloproteinases (MMPs) are present in virtually all areas of the eye and are responsible for the maintenance and remodeling of ocular architecture by influencing a wide range of processes such as basement membrane remodeling, blood–retinal barrier integrity, wound healing, debris clearance, neovascularization, and fibrotic repair tissue deposition.^1–3^ MMPs are believed to have a role in the pathophysiology of many ocular diseases including dry eye disease, age-related macular degeneration, diabetic retinopathy, and glaucoma (Table 1).^4^ This article reviews the role of MMPs in glaucoma, the involvement of MMPs in the mechanism of intraocular pressure (IOP) lowering with topical prostaglandin analog/prostamide (PGA) medications, and the development of a PGA intracameral implant to elicit more sustained MMP-mediated target tissue remodeling for an extended duration of effect.

**Table 1.** Evidence of Matrix Metalloproteinase Involvement in the Pathophysiology of Ocular Diseases

Disease or disorder	Experimental evidence of a role of MMPs
Age-related macular degeneration	Levels of MMP-2 and MMP-9 activity in Bruch's membrane-choroid tissue samples from patients with age-related macular degeneration were significantly lower than those in samples from control eyes^113^
Bacterial keratitis	Upregulation of MMP-9 expression in response to lipopolysaccharide exposure was more pronounced in primary corneal fibroblasts cultured from patients with bacterial keratitis compared with fibroblasts cultured from healthy controls; the upregulation of MMP-9 activity is proposed to be involved in corneal ulceration and perforation^114^
Choroidal neovascularization	Studies of the levels of MMPs in choroidal neovascular membranes from patients with age-related macular degeneration have reported conflicting results^115^
	In a laser-induced rat choroidal neovascularization model, inhibition of MMP-2, MMP-9, MMP-3, and MT-MMP-1 with the synthetic selective MMP inhibitor prinomastat reduced the formation of invading fibrovascular lesions^116^
Climatic droplet keratopathy	Levels of MMP-2 and MMP-9 were increased in tear samples from patients with climatic droplet keratopathy compared with normal control patients, suggesting that MMP-2 and -9 could potentially be involved in the corneal scarring that occurs in the disease^117^
Corneal neovascularization	Senescent fibroblasts that accumulate in the corneal stroma in a mouse model of alkali-induced corneal wound healing and neovascularization show increased expression of MMP-2, -3, and -14 relative to normal fibroblasts, and in normal mice, injection of hydrogen peroxide-induced premature senescent fibroblasts promoted corneal neovascularization that was blocked by a synthetic MMP inhibitor, GM6001^118^
Diabetic retinopathy	Preparations of microvessels from the retina of donors with diabetic retinopathy showed increased MMP-9 activity, and exposure to glucose stimulated MMP-9 expression in isolated bovine retinal endothelial cells^119^
	In a rat model of diabetic retinopathy, retinal levels of mRNA for MMP-2, MMP-9, and MMP-14 were increased; furthermore, exposure to MMP-2 and MMP-9 reduced tight junction function in cultures of bovine retinal endothelial cells, suggesting that these MMPs might have a role in the breakdown of the blood–retinal barrier in diabetic retinopathy^120^
Dry eye disease	Corneal epithelium damage in experimentally induced dry eye was attenuated in MMP-9 knockout mice compared with wild-type mice^15^
Keratoconus	Levels of MMP-1, -3, -7, and -13 and collagenase and gelatinase activities were increased in the tear film of patients with keratoconus compared with controls^121^
Pseudoexfoliation glaucoma	Levels of MMP-2 in the aqueous humor were positively correlated with the degree of chamber angle pigmentation and IOP^122^
Primary open-angle glaucoma	Levels of endogenously activated MMP-2 were significantly decreased in aqueous samples from primary open-angle glaucoma patients compared with cataract control patients^47^
	TIMP-2 levels were significantly increased in patients with primary open-angle glaucoma compared with cataract control patients^48^
Pterygium	Immunohistochemistry showed the presence of MMP-9, MMP-10, and TIMP-1 in 35%, 34%, and 72% of pterygial specimens taken from 82 patients^123^
	Inhibition of *TIMP-1* gene expression by siRNA in cultures of pterygium epithelial cells resulted in increased cellular activity in cellular migration and invasion assays^123^
Spontaneous autoimmune uveitis	MMP-2 and MMP-14 are decreased and MMP-9 is increased in retinal tissue and vitreous humor samples from animals with equine recurrent uveitis^124^
Superior limbic keratoconjunctivitis	Expression of MMP-1 and MMP-3 was increased in conjunctival specimens and cultured conjunctival fibroblasts from patients with superior limbic keratoconjunctivitis compared with controls^125^
Viral corneal ulcers	Polyinosinic-polycytidylic acid, a synthetic analog of viral double-stranded RNA, induced the expression and secretion of MMP-1 and MMP-3 by cultured human corneal fibroblasts; expression of MMPs by corneal fibroblasts could be responsible for collagen degradation in the corneal stroma, leading to corneal ulceration and perforation after viral infection^126^

IOP, intraocular pressure; MMP, matrix metalloproteinase; MT-MMP, membrane-type MMP; TIMP, tissue inhibitor of metalloproteinase.

## Overview of the Role of MMPs in Health and Disease

Tissues contain cells in a scaffold of extracellular matrix (ECM) composed predominantly of a heterogeneous and tissue-specific mix of fibrous proteins (predominantly collagen), proteoglycans, and glycosaminoglycans.^5^ The ECM is a highly dynamic structure that undergoes continuous remodeling to maintain cellular and tissue homeostasis.^5^ This remodeling is largely mediated by a large family of zinc-dependent proteolytic enzymes known as the MMPs, whose substrates include ECM components (eg, collagen, fibronectin), as well as secreted cytokines (eg, tumor necrosis factor-α, transforming growth factor-β) and cell adhesion molecules (eg, e-cadherin).^1^

**FIG. 1.**
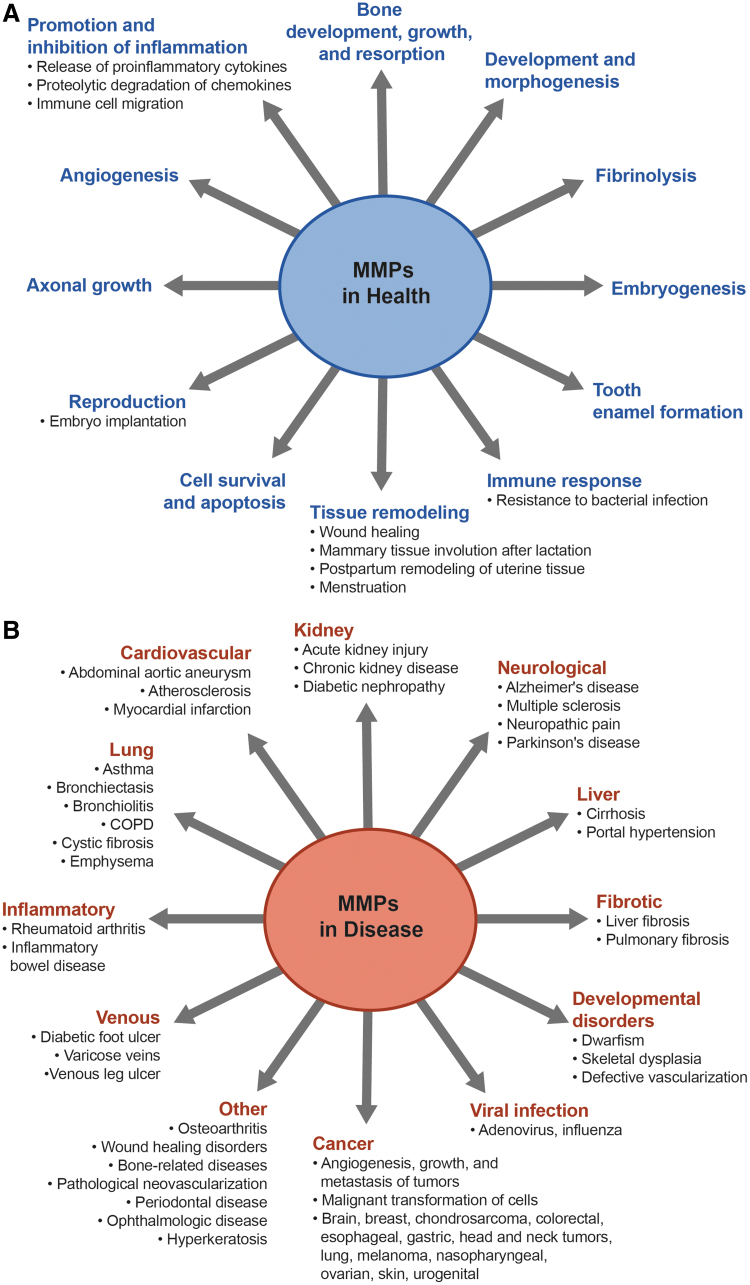
Role of MMPs in physiologic **(A)** and pathologic conditions **(B)**.^2,8,19,112^ MMP, matrix metalloproteinase.

MMP-mediated degradation of the ECM has an important role in growth and development, morphogenesis, and tissue repair and remodeling (Fig. 1A). For instance, over half of the members of the MMP family are involved in promoting bone growth and development under normal physiological conditions,^6^ and mutations in the human *MMP2* and *MMP14* genes have been linked to rare skeletal diseases characterized by loss of bone tissue and short stature.^7^

MMPs are produced by a wide variety of cell types (eg, stromal fibroblasts, macrophages, and endothelial and epithelial cells) and are expressed as inactive zymogens (proenzymes) containing a propeptide domain that must be removed for protease activity.^2,4,8^ In vertebrates, there are at least 28 different MMPs, and at least 23 of them are expressed in humans.^2^ Based on their substrate specificities and structural domains, MMPs can be categorized into one of 6 subgroups (Table 2): collagenases (MMP-1, -8, -13, -18); gelatinases (MMP-2 and MMP-9); stromelysins (MMP-3, -10, -11); matrilysins (MMP-7, -26); membrane-type matrix metalloproteinases (MT1/2/3/4/5/6-MMP or MMP-14, -15, -16, -17, -24, -25); and unclassified MMPs (MMP-12, -19, -20, -21, -23, -27, -28).^9^

**Table 2.** Members of the Matrix Metalloproteinase Family and Their Distribution and Substrates

MMP (alternative name)	Distribution	Collagen substrates	Noncollagen ECM substrates	Other targets and substrates
Collagenases				
MMP-1 (collagenase-1)	Endothelium, intima, smooth muscle cells, fibroblasts, vascular adventitia, platelets, varicose veins (interstitial/fibroblast collagenase)	I, II, III, VII, VIII, X, gelatin	Aggrecan, nidogen, perlecan, proteoglycan link protein, serpins, tenascin-C, versican	Casein, α1-antichymotrypsin, α1-antitrypsin, α1-proteinase inhibitor, IGF-BP-3 and -5, IL-1β, L-selectin, ovostatin, pro-TNF-α, SDF-1
MMP-8 (collagenase-2)	Macrophages, neutrophils (PMNL or neutrophil collagenase)	I, II, III, V, VII, VIII, X, gelatin	Aggrecan, elastin, fibronectin, laminin, nidogen	α2-Antiplasmin, proMMP-8
MMP-13 (collagenase-3)	Smooth muscle cells, macrophages, varicose veins, preeclampsia, breast cancer	I, II, III, IV, gelatin	Aggrecan, fibronectin, laminin, perlecan, tenascin	Casein, plasminogen activator 2, proMMP-9 and -13, SDF-1
MMP-18 (collagenase-4)^a^	Xenopus (amphibian, Xenopus collagenase) heart, lung, colon	I, II, III, gelatin		α1-Antitrypsin
Gelatinases				
MMP-2 (gelatinase-A, type IV collagenase)	Endothelium, VSM, adventitia, platelets, leukocytes, aortic aneurysm, varicose veins	I, II, III, IV, V, VII, X, XI, gelatin	Aggrecan, elastin, fibronectin, laminin, nidogen, proteoglycan link protein, versican	Active MMP-9 and -13, FGF-R1, IGF-BP-3 and -5, IL-1β, pro-TNF-α, TGF-β
MMP-9 (gelatinase-B, type IV collagenase)	Endothelium, VSM, adventitia, microvessels, macrophages, aortic aneurysm, varicose veins	IV, V, VII, X, XIV, gelatin	Aggrecan, elastin, fibronectin, laminin, nidogen, proteoglycan link protein, versican	CXCL5, IL-1β, IL2-R, plasminogen, pro-TNF-α, SDF-1, TGF-β
Stromelysins				
MMP-3 (stromelysin-1)	Endothelium, intima, VSM, platelets, coronary artery disease, hypertension, varicose veins, synovial fibroblasts, tumor invasion	II, III, IV, IX, X, XI, gelatin	Aggrecan, decorin, elastin, fibronectin, laminin, nidogen, perlecan, proteoglycan, proteoglycan link protein, versican	Casein, α1-antichymotrypsin, α1-proteinase inhibitor, antithrombin III, E-cadherin, fibrinogen, IGF-BP-3, L-selectin, ovostatin, pro-HB-EGF, pro-IL-1β, proMMP-1, -8, and -9, pro-TNF-α, SDF-1
MMP-10 (stromelysin-2)	Atherosclerosis, uterus, preeclampsia, arthritis, carcinoma cells	III, IV, V, gelatin	Aggrecan, elastin, fibronectin, laminin, nidogen	Casein, proMMP-1, -8, and -10
MMP-11 (stromelysin-3)	Brain, uterus, angiogenesis	Does not cleave	Aggrecan, fibronectin, laminin	α1-Antitrypsin, α1-proteinase inhibitor, IGF-BP-1
Matrilysins				
MMP-7 (matrilysin-1)	Endothelium, intima, VSM, uterus, varicose veins (PUMP)	IV, X, gelatin	Aggrecan, elastin, enactin, fibronectin, laminin, proteoglycan link protein	Casein, β4 integrin, decorin, defensin, E-cadherin, Fas-ligand, plasminogen, proMMP-2, -7, and -8, pro-TNF-α, syndecan, transferrin
MMP-26 (matrilysin-2, endometase)	Breast cancer, endometrial tumors	IV, gelatin	Fibrinogen, fibronectin, vitronectin	Casein, β1-proteinase inhibitor, fibrin, fibronectin, proMMP-2
Membrane type				
MMP-14 (MT1-MMP)	VSM, fibroblasts, platelets, brain, uterus, angiogenesis	I, II, III, gelatin	Aggrecan, elastin, fibrin, fibronectin, laminin, nidogen, perlecan, proteoglycan, tenascin, vitronectin	α_v_β_3_ integrin, CD44, proMMP-2 and -13, pro-TNF-α, SDF-1, α1-proteinase inhibitor, tissue transglutaminase
MMP-15 (MT2-MMP)	Fibroblasts, leukocytes, preeclampsia	I, gelatin	Aggrecan, fibronectin, laminin, nidogen, perlecan, tenascin, vitronectin	ProMMP-2 and -13, tissue transglutaminase
MMP-16 (MT3-MMP)	Leukocytes, angiogenesis	I	Aggrecan, fibronectin, laminin, perlecan, vitronectin	Casein, proMMP-2 and -13
MMP-17 (MT4-MMP)	Brain, breast cancer	Gelatin	Fibrin	
MMP-24 (MT5-MMP)	Leukocytes, lung, pancreas, kidney, brain, astrocytoma, glioblastoma	Gelatin	Chondroitin sulfate, dermatan sulfate, fibrin, fibronectin, N-cadherin	ProMMP-2 and -13
MMP-25 (MT6-MMP)	Leukocytes (leukolysin), anaplastic astrocytomas, glioblastomas	IV, gelatin		Fibrin, fibronectin, proMMP-2, α1-proteinase inhibitor
Other MMPs				
MMP-12 (metalloelastase)	Smooth muscle cells, fibroblasts, macrophages, great saphenous vein	IV, gelatin	Elastin, fibronectin, laminin	Casein, plasminogen
MMP-19 (RASI-1)	Liver	I, IV, gelatin	Aggrecan, fibronectin, laminin, nidogen, tenascin	Casein
MMP-20 (enamelysin)	Tooth enamel	V	Aggrecan, cartilage oligomeric protein, amelogenin	
MMP-21 (Xenopus-MMP)	Fibroblasts, macrophages, placenta			α1-Antitrypsin
MMP-23 (CA-MMP)	Ovary, testis, prostate	Gelatin		
	Other (type II) MT-MMP			
MMP-27 (human MMP-22 homolog)	Heart, leukocytes, macrophages, kidney, endometrium, menstruation, bone, osteoarthritis, breast cancer			
MMP-28 (epilysin)	Skin, keratinocytes			Casein

Adapted from Cui et al.,^2^ Copyright 2016, with permission from Elsevier.

^a^ Not found in humans.

CA-MMP, cysteine array MMP; CXCL5, chemokine (C-X-C motif) ligand 5; ECM, extracellular matrix; FGF-R1, fibroblast growth factor receptor 1; IGF-BP, insulin-like growth factor binding protein; IL, interleukin; MMP, matrix metalloproteinase; MT-MMP, membrane-type MMP; PMNL, polymorphonuclear leukocytes; pro-HB-EGF, pro-heparin-binding epidermal growth factor-like growth factor; RASI-1, rheumatoid arthritis synovium inflamed-1; SDF-1, stromal cell-derived factor 1; TGF, transforming growth factor; TNF, tumor necrosis factor; VSM, vascular smooth muscle.

Many of the MMPs are secreted as proenzymes and are activated extracellularly.^2^ However, MMP-11, -21, and -28 and the MT-MMPs have a furin-like proprotein convertase recognition sequence at the C-terminus of the propeptide, and these proenzymes are activated intracellularly by furin.^2^ After the MT-MMPs are activated intracellularly, they are transported to the cell surface where they can cleave and activate other proMMPs.^2^

During normal steady-state conditions, levels of MMPs in most tissues are low or negligible.^10^ MMP expression is transcriptionally controlled by inflammatory cytokines, growth factors, hormones, and cell–cell and cell–matrix interactions. Activation of the proenzyme to the active form can be mediated by endopeptidases such as furin, serine proteases, or other MMPs.^8^ For example, MT1-MMP can activate proMMP-2.^11^ Activity of the MMPs is further regulated by 2 major types of endogenous inhibitor, α2-macroglobulin and tissue inhibitors of metalloproteinase (TIMPs).^4,10^ The α2-macroglobulin inhibitors are confined to the blood and lymph and result in irreversible clearance of MMPs through endocytosis of α2-macroglobulin/MMP complexes.^12^ Four homologous TIMPs (TIMP-1, -2, -3, and -4) have been identified; each binds MMPs in a 1:1 stoichiometry and leads to reversible inhibition of MMPs.^12^ Each TIMP can inhibit multiple MMPs with varying specificity and affinity,^2,9^ and the ratio of MMP:TIMP often determines the extent of ECM turnover.^2^

Alterations in MMP expression and activity occur in normal biological processes (eg, wound healing), but imbalances in the ratio of MMP and TIMP levels can cause abnormal tissue remodeling due to the excessive degradation or accumulation of ECM components and changes in growth factors, receptor signaling, and cell migration.^2,13^ Such imbalances contribute to pathological conditions such as cardiovascular disease (eg, atherosclerosis), musculoskeletal disorders (eg, arthritis), neurodegenerative diseases (eg, Alzheimer's, Parkinson's), and various cancers (Fig. 1B).^2,14^

Evidence suggests that MMPs are involved in the inflammation, angiogenesis, and degenerative processes that occur in several ocular diseases. For example, levels and activity of MMP-9 in the tear film have been shown to be elevated in patients with dry eye disease, and studies of experimentally induced dry eye in MMP-9 knockout and wild-type mice have demonstrated that MMP-9 participates in the destruction of the corneal epithelium caused by dry eye.^15^ Studies using MMP-2 and MMP-9 knockout and wild-type mice have further implicated both MMP-2 and MMP-9 in the development of choroidal neovascularization induced by laser-induced rupture of Bruch's membrane in a mouse model of neovascular age-related macular degeneration.^16^

Efforts to exploit MMPs in the treatment of disease typically have focused on inhibiting MMP activity. Broad-spectrum synthetic inhibitors of MMPs have been tested in various types of cancer, and use of inhibitors of specific MMPs remains a viable potential strategy to reduce tumor metastasis.^17,18^ MMP inhibition also has been proposed as a therapeutic approach for diabetic foot ulcers and currently is utilized in the treatment of periodontal disease.^19^ In contrast, PGAs that increase MMP activity have proven to be useful in the treatment of glaucoma. Glaucoma is treated by lowering IOP, and increasing MMP activity in target intraocular tissues for IOP lowering causes ECM remodeling that results in a decrease in IOP.

## Glaucoma, Aqueous Outflow Pathways, and MMPs

Glaucoma is a progressive optic neuropathy and the leading cause of irreversible blindness. It is estimated to affect 3.5% of the global population aged between 40 and 80 years.^20^ IOP is the major modifiable risk factor for the onset and progression of glaucoma, and there is overwhelming evidence that reducing IOP has a protective effect on the visual field in glaucoma, even when IOP is already in the normal range.^21–23^ As a consequence, treatment modalities for glaucoma, whether surgical or pharmaceutical, are aimed at lowering IOP.

**FIG. 2.**
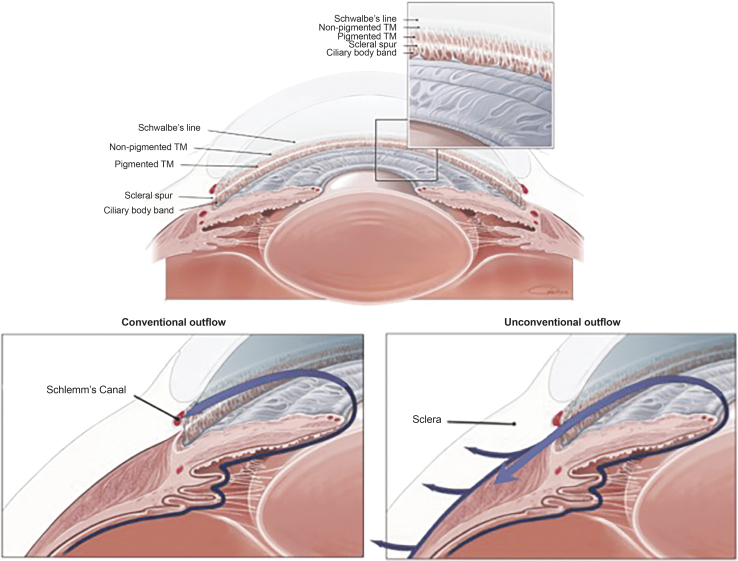
Anatomy of the eye and aqueous humor outflow pathways. Aqueous humor is produced by the ciliary body and moves through the pupil into the anterior chamber where it exits the eye. In the conventional outflow pathway, the aqueous filters through the TM into the lumen of Schlemm's canal, which drains into aqueous veins and the episcleral venous system. In the unconventional outflow pathway, aqueous drains through the ciliary muscle, supraciliary and suprachoroidal spaces, and sclera. TM, trabecular meshwork.

Normal IOP ranges between 10 and 21 mm Hg^24^ and is maintained through regulation of the production and outflow of aqueous humor, which supplies oxygen and glucose to the avascular lens, cornea, iris, and trabecular meshwork (TM) of the eye. Although the secretion of aqueous humor by the ciliary body and its drainage through the unconventional outflow pathways (uveoscleral and uveovortex) are relatively pressure insensitive, outflow through the conventional (trabecular) pathway is pressure dependent, and IOP is determined in large part by the resistance generated in the conventional outflow pathway (Fig. 2), where the majority of aqueous humor exits the eye.^25–28^ An early study using a tracer method to directly measure uveoscleral outflow in 2 human eyes reported that <15% of the total aqueous outflow occurred through the uveoscleral pathway.^29^ Although studies using indirect methods to assess unconventional outflow have varied widely, they most frequently have reported that it accounts for 25%–50% of the total aqueous outflow in human eyes.^28^ Disparate findings among and within studies have been attributed to differing methodologies (use of tonographic or fluorophotometric measurement of outflow facility, and assumed episcleral venous pressure) and to an age-related decrease in unconventional outflow.^28,30^

The TM is a porous tissue with 2 regions involved in filtering aqueous with minimal impedance: the uveal meshwork and the corneoscleral meshwork.^31^ In contrast, the third region of the TM, the deepest one, the juxtacanalicular tissue (also known as the cribriform region) generates outflow resistance through interactions with the inner wall of Schlemm's canal.^27^ The uveal and corneoscleral meshworks are composed of connective tissue beams and sheets/lamellae, with large intertrabecular spaces between adjacent sheets.^32^ Aqueous humor drains through the intratrabecular spaces of the TM and the loosely arranged ECM of the juxtacanalicular tissue and is removed through the Schlemm's canal into collector channels, and from there into aqueous veins and the episcleral venous system.^31,32^ Increased IOP in primary open-angle glaucoma is caused by an increase in aqueous humor outflow resistance, measured as a decrease in tonographic outflow facility,^33^ which leads to a decrease in pressure-sensitive outflow. Enhancing outflow through the TM pathway is a recognized approach for lowering IOP in glaucoma, both to correct the deficit that causes elevated IOP and to allow the eye to recover from transient pressure disturbances (eg, increased pressure from a pillow pressing against the eye during sleep).^33^

The ECM of the juxtacanalicular region participates in the generation of resistance to aqueous humor outflow through the TM pathway.^31^ The ECM of the TM consists of collagens, laminins, elastin, fibronectin, fibrillins, and proteoglycans^34^ and is continuously being remodeled by members of the MMP family (eg, MMP-1, -2, -3, -9, -12, and -14).^12^ Studies using perfused human anterior segment organ cultures and human TM cell cultures have shown that increases in IOP cause an increase in MMP-2 expression, and mechanical stretching of TM cells causes an increase in MMP-2 expression and a decrease in TIMP-2 expression.^35^ These results support a hypothesis of a feedback mechanism for IOP homeostasis,^35^ in which TM cells sense an increase in IOP through stretch/distortion of the ECM, and they respond by increasing MMP and decreasing TIMP expression. This results in enhanced ECM turnover, which causes a decrease in the TM resistance to aqueous humor outflow and a decrease in IOP, restoring IOP to normal levels.

Early evidence for MMPs producing ECM remodeling that enhances aqueous humor outflow through the TM was provided by a study using perfused human anterior segment organ cultures.^36^ The addition of recombinant MMP-2, MMP-3, or MMP-9 resulted in a reversible increase in outflow facility, whereas inhibition of endogenous MMP activity reduced outflow facility.^36^ More recently, altered structural organization of the TM and early-onset ocular hypertension were observed in MMP-9 knockout mice, suggesting that remodeling of the TM by MMP-9 is needed to enhance outflow and maintain IOP homeostasis.^37^

**FIG. 3.**
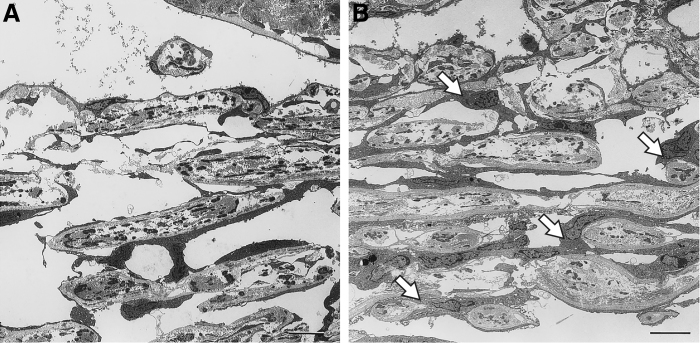
Electron micrographs of the TM of primates treated for 1 year with placebo **(A)** or systemic corticosteroid **(B)**. The control animals showed normal TM morphology. In contrast, the TM in animals treated with corticosteroid demonstrated an increase in ECM in the juxtacanalicular region. This accumulation of ECM increases the hydraulic resistance to aqueous outflow leading to elevated IOP. Scale bars, 10 μm. *Arrows* show increased ECM. Republished from Clark et al.^41^ with permission. © ARVO (Association for Research in Vision & Ophthalmology). ECM, extracellular matrix. IOP, intraocular pressure.

Studies of the mechanisms of steroid-induced increased IOP and glaucoma have also provided evidence of the involvement of MMPs in IOP regulation. Enhanced juxtacanalicular deposition of fibrous material has been observed with electron microscopy in tissue samples from patients with corticosteroid-induced glaucoma.^38^ The accumulation of ECM components as a contributing mechanism in steroid-induced glaucoma was supported by the findings from studies investigating the effects of corticosteroids in cell cultures^39,40^ and animal models.^41,42^ Exposure of TM organ cultures to corticosteroids led to a decrease in extracellular protease activities, including 92 kDa type IV collagenase activity (MMP-9).^39^ In another study using human corneoscleral explant cultures containing both ciliary body and TM, exposure to dexamethasone led to decreased activity of MMP-2, MMP-3, and MMP-9 on zymography.^40^ Long-term treatment with a corticosteroid in a primate model resulted in structural changes in the TM consistent with steroid-induced glaucoma, including increased ECM deposition in the juxtacanalicular region^41^ (Fig. 3). Finally, in a sheep model of steroid-induced ocular hypertension, gene therapy with a vector carrying an inducible MMP-1 human gene protected against and reversed corticosteroid-induced IOP increases.^42^ Together these findings suggest that corticosteroids downregulate MMPs in the TM (and possibly also in the ciliary body), leading to ECM deposition and tissue remodeling that reduces aqueous outflow and leads to increased IOP.

Uveoscleral outflow involves the resorption of aqueous humor through the intercellular spaces of the ciliary muscle into the suprachoroidal space, with transscleral flow occurring through interfibrillary and perivascular spaces of the scleral stroma.^43^ Compaction of the scleral ECM affects transscleral permeability.^44^ As collagen accounts for 75% of the total dry weight of sclera,^44^ collagen density within the sclera can be expected to affect permeability,^45^ and MMP collagenase activity in the sclera is very likely to cause tissue remodeling that enhances transscleral outflow. In addition, immunohistochemistry studies have shown the presence of MMP-1 in tissues associated with the uveoscleral outflow pathway in normal human eyes (ciliary muscle, iris root, and sclera), suggesting that MMP-1 activity may be a key regulator of uveoscleral outflow.^46^

In a study evaluating aqueous humor concentrations of MMPs and TIMPs, levels of endogenously activated MMP-2 were reported to be significantly decreased in aqueous samples from primary open-angle glaucoma patients compared with cataract control patients, while levels of TIMP-2 were unchanged.^47^ In contrast, in a similar study that evaluated aqueous humor concentrations of MMP-2 and TIMP-2, TIMP-2 levels were significantly increased in patients with primary open-angle glaucoma compared with cataract control patients.^48^ Importantly, the results of both studies suggest that an imbalance in the MMP/TIMP ratio may contribute to the pathogenesis of decreased outflow facility and elevated IOP in glaucoma.^47,48^

## Topical PGAs in Glaucoma Management

The first topical PGA for IOP lowering, latanoprost, was introduced in the 1990s. Over the past 2 decades, topical PGAs have become commonly used first-line glaucoma therapies because they reduce IOP more effectively than other classes of topical ocular hypotensive medications^49^ and are well tolerated and systemically safe.^50^ Common side effects include conjunctival hyperemia, eyelash changes, and hyperpigmentation of the iris and periocular skin.^50^ PGA eye drops, which are instilled once daily, include prostaglandin (PG) ester prodrugs (latanoprost, travoprost, tafluprost) and the prostamide, bimatoprost. The prodrugs are quantitatively hydrolyzed by esterases in the cornea into their biologically active free acid forms.^51^ Bimatoprost is less efficiently hydrolyzed by corneal amidases.^51^ Both the intact bimatoprost molecule and bimatoprost free acid have biological activity^52–54^ and are present in ocular tissues after topical bimatoprost administration,^51^ and both the intact bimatoprost molecule and the free acid have been proposed to contribute to the IOP-lowering effects of topical bimatoprost.^51,55–58^

The PGAs lower IOP by enhancing aqueous outflow,^59^ and latanoprost, bimatoprost, and travoprost consistently have been reported to increase outflow through the uveoscleral pathway.^60^ However, the methods used for measurement of outflow facility in human eyes are imprecise and have influenced findings of the relative contribution of enhanced pressure-sensitive and pressure-insensitive outflow to the IOP lowering produced by topical PGAs.^59^ Bimatoprost has been reported to have a dual mechanism of action, reducing IOP by enhancing both pressure-insensitive (presumed uveoscleral) and pressure-sensitive (presumed TM) outflow. The enhancement of pressure-sensitive outflow was primarily responsible for the reduction of IOP produced by topical bimatoprost in normal subjects.^33^

On a molecular level, the enhancement of aqueous outflow by PGAs is believed to involve MMP-mediated increased turnover of the ECM in the uveoscleral and TM aqueous outflow pathways, leading to reduced outflow resistance.^60^

## Evidence Implicating MMPs in the Mechanism of Action of Topical PGAs

An early study from the 1980s using cynomolgus monkeys^61^ suggested that ECM turnover in the ciliary body was critically involved in the mechanism of IOP lowering with topical PGAs.^61^ The increase in uveoscleral outflow facility produced by PGA treatment was associated with pronounced changes in extracellular material within the ciliary muscle (Table 3), which were suspected to result from collagenolytic activity described with PGAs in other organs. A study using human ciliary smooth muscle cells exposed to PGF2α for 3 days provided supporting evidence that MMPs were involved in the ECM turnover: concentrations of proMMP-1 and -3 in the culture medium were increased by 254% and 128%, respectively, and similar increases were observed after exposure to other PGs.^62^

**Table 3.** Key Studies of the Effects of Prostaglandin-Related Compounds on Matrix Metalloproteinase Expression and the Morphology of Aqueous Humor Outflow Pathways

Study	PG or PGA	Experimental model	Duration of exposure	Effects
Uveoscleral tissues				
Lütjen-Drecoll et al.^61^	PGF2α tromethamine salt, PGF2α isopropyl ester	Cynomolgus monkeys	Once or twice daily topical treatment for 4–8 days	Ciliary muscle showed empty spaces between muscle fiber bundles, and loss of reticular fibers and ground substance in the enlarged spaces could be responsible for the increase in uveoscleral outflow
Lindsey et al.^62^	PGF2α, 17-phenyltrinor-PGF2α, 11-deoxy-PGE_1_	Human ciliary smooth muscle cells	Exposure for 1–3 days	Increased proMMP-1 and -3 concentrations in the culture medium
Weinreb et al.^63^	PGF2α, 17-phenyltrinor-PGF2α, 11-deoxy-PGE_1_, latanoprost acid	Human ciliary smooth muscle cells	Exposure for 1–3 days	Increased concentration of MMP-1, -2, -3, and -9 in the culture medium
Sagara et al.^127^	PGF2α-isopropyl ester	Cynomolgus monkeys	Twice daily topical treatment for 5 days	Significant reductions in scleral collagen (most notably type I and III) immunoreactivity in the ciliary muscle and adjacent sclera
Kim et al.^64^	Latanoprost acid, PGF2α, 17-phenyltrinor-PGF2α	Human scleral organ cultures	Exposure for 24, 48, or 72 h	Increased transscleral permeability accompanied by increased MMP expression (MMP-2 > MMP-3 > MMP-1)
Gaton et al.^65^	PGF2α-isopropyl ester	Cynomolgus monkeys	Twice daily topical treatment for 5 days	Significant increase in MMP (-1, -2, and -3) expression in tissues of uveoscleral outflow pathway
Weinreb and Lindsey^66^	Latanoprost acid	Human ciliary smooth muscle cells	Exposure to concentrations of 8–1,000 nM for 24 h	Concentration-dependent increases in MMP-1, -3, and -9 mRNA levels
Anthony et al.^67^	Latanoprost acid	Human ciliary smooth muscle cells	Exposure to concentrations of 1–1,000 nM for 6, 18, or 24 h	Concentration- and time-dependent increase in TIMP-1 protein and TIMP-1 mRNA levels, brief and minor increase in TIMP-2 protein
Richter et al.^76^	Bimatoprost 0.03%, latanoprost 0.005%, sulprostone 0.03%, AH13205 0.1%	Cynomolgus monkeys	Topical treatment for 1 year	Enlarged and more organized spaces between muscle bundles of ciliary muscle for outflow; doubling in number of nerve fiber bundles in ciliary muscle
Hinz et al.^128^	Latanoprost acid	Human nonpigmented ciliary epithelial cells	Exposure for 24 h	Increase in COX-2 mRNA expression leading to increased levels of PGE2 in culture medium and increased expression of MMP-1 mRNA
Oh et al.^68^	Latanoprost acid	Human ciliary body tissue and smooth muscle cells	Exposure for 24 h	Upregulation of MMP-3, -9 (low expression), -17, TIMP-3 mRNA expression; downregulation of MMP-1, -2, -12, -14, -15, -16, TIMP-4 mRNA expression
Ooi et al.^86^	Bimatoprost acid, latanoprost acid, unoprostone acid	Human ciliary body smooth muscle cells isolated from donor corneoscleral rims	Exposure for 24 h	The different PGAs produced different ratios of MMP/TIMP, potentially related to their differences in intraocular pressure-lowering efficacy; for example, all PGAs increased MMP-1, -3, and -9; MMP-2 levels were decreased by unoprostone and unaffected by bimatoprost and latanoprost; and all PGAs increased TIMP-3, but only unoprostone increased TIMP-1 and -4
Trabecular meshwork and aqueous humor				
Richter et al.^76^	Bimatoprost 0.03%, latanoprost 0.005%, sulprostone 0.03%, AH13205 0.1%	Cynomolgus monkeys	Topical treatment for 1 year	Disconnection of some endothelial cells of the inner wall of Schlemm's canal from the subendothelial layer, with loss of ECM underneath the endothelium and through the juxtacanalicular region
Oh et al.^69^	Latanoprost acid	Human TM tissue and endothelial cells	Exposure for 24 h	Increase in mRNA expression of MMP-1, -3, -17, -24 and decrease in mRNA expression of MMP-11 and -15; upregulation of TIMP-2, -3, -4
Wan et al.^58^	Bimatoprost	Human anterior segments and TM cells		Increase in outflow facility by 40% on average; increase in hydraulic conductivity of trabecular meshwork cell monolayers by 78%
Bahler et al.^70^	Latanoprost acid, PGE1	Cultured human anterior segments, including TM and Schlemm's canal	Continuous infusion up to 72 h	Focal detachment and loss of Schlemm's canal cells; no consistent change in MMP-2, -3, or -9 activity in the anterior segments
Yamada et al.^87^	Bimatoprost acid, latanoprost acid, tafluprost acid	Human nonpigmented ciliary epithelial cell cultures	Exposure for 24 h	Each PGA induced a concentration-dependent increase in mRNA levels for MMP-1, -2, -3, -9, and -17 and decrease in mRNA levels for TIMP-1 and -2

Adapted from Toris et al.^60^ Update on the Mechanism of Action of Topical Prostaglandins for Intraocular Pressure Reduction, S107–120, Copyright © 2008, with permission from Elsevier.

COX-2, cyclooxygenase-2; ECM, extracellular matrix; MMP, matrix metalloproteinase; TIMP, tissue inhibitor of metalloproteinase; TM, trabecular meshwork; PG, prostaglandin; PGA, prostaglandin analog/prostamide.

**FIG. 4.**
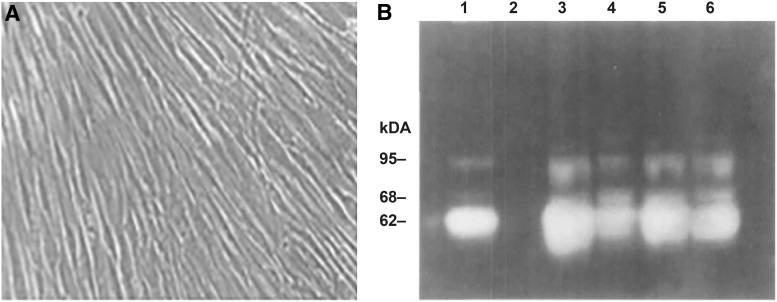
Increase in MMP activity in the culture medium of human ciliary muscle cells after PG exposure. **(A)** Third-passage human ciliary muscle cells from a 59-year-old eye form a monolayer with aligned spindle-shaped cells. **(B)** Gelatin zymogram of media from cells exposed for 72 h to vehicle control (lane 1) or to 200 nM PGF2α (lane 3), 17-phenyltrinor-PGF2α (lane 4), 11-deoxy-PGE1 (lane 5), or latanoprost acid (lane 6). Band sizes increased in medium from PG-treated cultures, indicating increased MMP activity. Used with permission of ARVO, from Weinreb et al.^63^; permission conveyed through Copyright Clearance Center, Inc. PG, prostaglandin.

In a follow-up study, cultured human ciliary smooth muscle cells were incubated with latanoprost acid, PGF2α, 17-phenyltrinor-PGFα, or 11-deoxy-PGE_1_ (Fig. 4 and Table 3).^63^ MMP protease activity in the medium was assayed using gelatin and casein zymography, and Western blot results confirmed that the bands in the zymographs were MMP-1, -2, -3, and -9. Exposure to the PGs for 72 h increased levels of activity for all 4 MMPs compared with vehicle, confirming that MMP release is altered by exposure to PGs (Fig. 4).^63^ To further elucidate the effect of PGs on MMPs and its relationship to changes in uveoscleral outflow, these investigators incubated human scleral tissue with PGs and measured scleral permeability by evaluating perfusion of dextrans across the scleral tissue with a 2-chamber Ussing apparatus.^64^ PGF2α and PGAs (including latanoprost acid) increased tissue permeability in a time- and concentration-dependent manner, and the increase in permeability was associated with increases in the expression of MMPs (MMP-2 > MMP-3 > MMP-1) as measured by an enzyme-linked immunosorbent assay.^64^

Evidence of alterations in MMP expression, associated with IOP lowering in response to a topical PGA, was subsequently confirmed in primates.^65^ Immunoreactivity for MMPs (-1, -2, and -3) in iris root, ciliary muscle, and adjacent sclera was significantly increased after topical treatment of cynomolgus monkeys with PGF2α-isopropyl ester for 5 days in comparison with contralateral vehicle-treated eyes.^65^ Importantly, the intensity of immunostaining for MMP-2 in the ciliary muscle was significantly associated with the magnitude of the IOP reduction.^65^

The same group also investigated whether the PGA-induced increased MMP expression by ciliary body muscle cells was preceded by changes in MMP mRNA levels, suggesting transcriptional regulation of MMP expression, as had been reported in other cell types.^66^ Total RNA was isolated from cultured human ciliary smooth muscle cells, and the expression of mRNAs for MMP-1, -2, -3, and -9 was determined using real-time polymerase chain reaction (RT-PCR) assays. Latanoprost acid induced a concentration-dependent upregulation of MMP-1 (3- to 13-fold), -3, and -9 mRNA levels and no change in MMP-2 mRNA levels compared with mRNA levels in vehicle-treated cultures.^66^

In another study using cultured ciliary muscle cells, the induction of MMP-1 and -2 expression and activity by latanoprost acid was found to be accompanied by a simultaneous induction of TIMP-1.^67^ Concentration- and time-dependent increases in TIMP-1 protein appeared on Western blot analysis of human ciliary muscle cells incubated with latanoprost acid, and TIMP-1 mRNA levels were also upregulated.^67^ Induction of TIMP mRNA with latanoprost acid was corroborated in a subsequent study in which MMP and TIMP mRNA levels were evaluated with a long-term goal of identifying those that are associated with the observed increased uveoscleral outflow.^68^ Latanoprost acid effects on MMP and TIMP mRNA expression in cultures of ciliary smooth muscle cells from human donors varied somewhat by donor, but exposure to latanoprost acid induced MMP-9 mRNA expression and caused increased expression of mRNA for MMP-3 (in 3 of 5 cultures), MMP-17 (in 4 of 5 cultures), and TIMP-3 (in all 5 cultures) and decreased expression of mRNA for MMP-1 (in all 5 cultures), MMP-2 (in 3 of 5 cultures), and TIMP-4 (in 2 of 5 cultures).^68^ The authors suggested that coordinated expression of MMP-3, -9, and -17 along with TIMP-3 might mediate the alteration of the ECM with latanoprost treatment.^68^

The effects of PGA treatment on MMP and TIMP expression in the TM, as well as the ciliary body, were investigated in a study using human corneoscleral explant cultures.^40^ After 72 h of incubation with latanoprost, levels of MMP-2, MMP-3, and MMP-9 in the medium were increased by 36%, 112%, and 156%, respectively, as measured by zymography. Immunohistochemistry showed increased staining for the MMPs after latanoprost exposure in the ciliary body, but not in the TM. No increase in staining for TIMP-1 or TIMP-2 was evident in either tissue.^40^ In a later study evaluating the effect of latanoprost on MMPs and TIMPs in the TM, mRNA for several MMPs (MMP-1, -2, -3, -11, -12, -14, -15, -16, -17, -19, and -24) and TIMPs (-1 to -4) was identified using RT-PCR in human TM tissue and in cultures of TM cells from human donors.^69^ Exposure of the cultures from some donors to latanoprost acid caused increased expression of mRNA for MMP-1, -3, -17, and -24 and TIMP-2, -3, and -4. The authors suggested that TIMP upregulation with latanoprost acid might possibly occur to compensate for the increase in MMPs.^69^ In contrast, in a study using 6 different TM cell strains isolated from human donor eyes, exposure for 24 h to a high concentration of bimatoprost caused increased expression of mRNA for MMP-1, MMP-10, MMP-11, MMP-14, and MMP-16 and decreased expression of TIMP-3 mRNA (Stamer et al. International Society for Eye Research/BrightFocus Foundation 2nd Glaucoma Symposium, Oct. 23–26, 2019; Atlanta, GA).

The results from the cell culture studies suggest that PGA exposure affects the expression of MMPs by TM cells, although the response among cell strains is variable. In 2 separate studies using perfused human anterior segments in organ culture to test conventional outflow separately from the uveoscleral pathway, bimatoprost^58^ and latanoprost acid and PGE1^70^ were found to significantly increase conventional outflow facility. In the first study, bimatoprost gradually increased outflow facility over 2 days of exposure,^58^ suggesting the possibility that the change in outflow facility might be mediated by changes in the ECM in the juxtacanalicular region. In the second study, histological assessments of the tissue after latanoprost acid treatment demonstrated significant remodeling characterized by a focal loss of Schlemm's canal endothelial cells as they were lifted off the basal lamina, consistent with loosening of the focal adhesions between the cells and the ECM.^70^ In 2 of the organ cultures, the endothelial cell loss was accompanied by focal loss of underlying ECM in the juxtacanalicular tissue.^70^ Although MMPs evaluated by Western blot, zymography, and immunohistochemistry were not consistently increased after latanoprost acid treatment of the organ cultures, and the early onset of the increase in outflow facility suggested that a mechanism in addition to upregulation of MMPs could be involved, one of 10 pairs of anterior segments showed an increase in MMP-3 on both Western blots and zymography.^70^

Upregulation of MMP activity and ECM turnover in the TM similarly has been implicated in the increase in outflow facility and decrease in IOP produced by adenosine A1 agonists.^71,72^ For example, trabodenoson, an adenosine mimetic with A1 selectivity, has been shown to increase MMP-2 activity and decrease fibronectin and collagen IV levels in 3-dimensional human TM cell cultures and has also been shown to increase outflow facility and lower IOP in young and aged mice.^72^ In addition, the ADAMTS (a disintegrin and metalloproteinase with thrombospondin motifs) family of extracellular proteases, related to the MMP family, also remodels the ECM,^73^ and treatment with recombinant ADAMTS-4 has been shown to increase outflow facility in cultured porcine and human anterior segments.^74^ ADAMTSs potentially could be involved in the mechanism of IOP lowering by PGAs downstream of MMP upregulation, as MMP-17 has been shown to activate ADAMTS-4.^75^

**FIG. 5.**
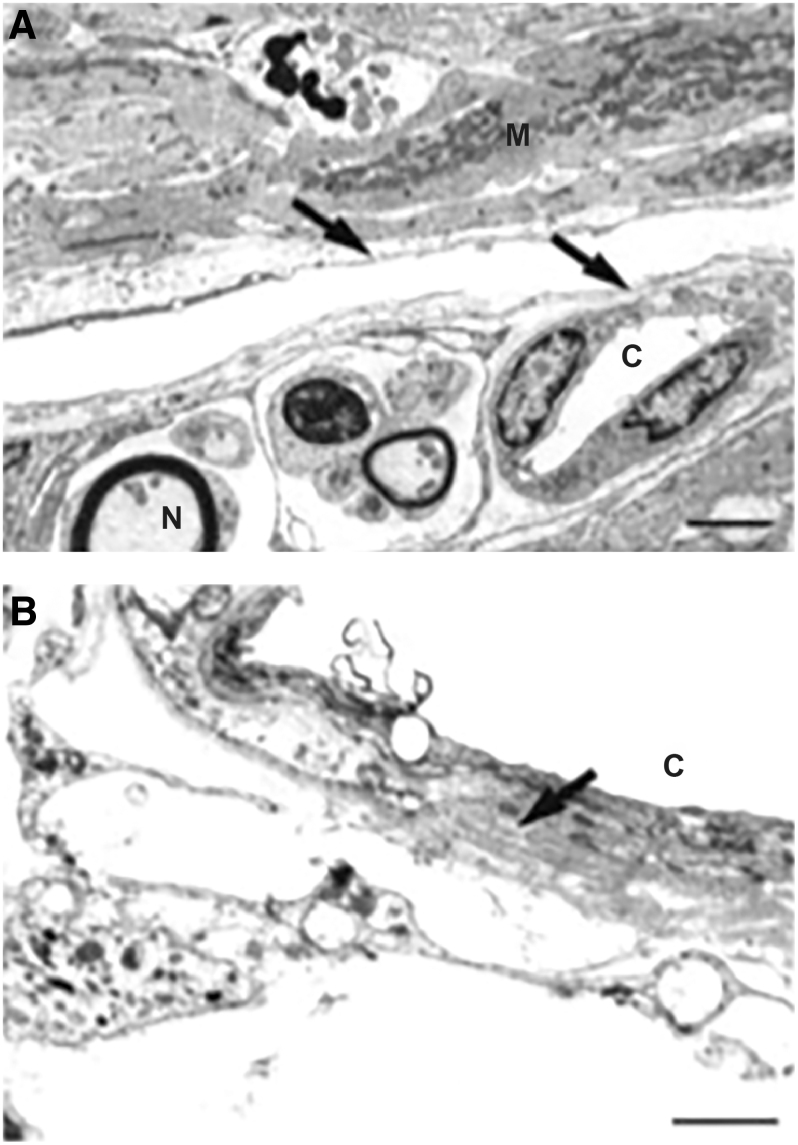
Electron micrographs of sagittal sections through the anterior longitudinal portion of the ciliary muscle of cynomolgus monkeys after 1 year of topical treatment with bimatoprost. Ciliary body remodeling after bimatoprost treatment included enlarged spaces for outflow between muscle bundles in the anterior ciliary muscle that were partially lined with endothelial-like cells **(***arrows* in **A)**. Capillaries within the enlarged intermuscular spaces had a thickened basement membrane **(***arrow* in **B)** in contact with some endothelial-like cells. Used with permission of ARVO, from Richter et al.^76^; permission conveyed through Copyright Clearance Center, Inc., Scale bars: **(A)** 2 μm; **(B)** 1 μm. C, capillary; M, muscle fiber bundles; N, nerve fiber.

Morphological changes indicative of ECM remodeling in both the ciliary muscle and TM were demonstrated in cynomolgus monkeys after long-term treatment with PGAs. Primate eyes were treated with topical bimatoprost, latanoprost, an EP2 agonist (AH13205), an EP3/EP1 agonist, or vehicle at IOP-lowering doses for 1 year.^76^ Sections from the 4 quadrants of the circumference of the eyes were investigated qualitatively and quantitatively using light and electron microscopy. Tissue remodeling was apparent and similar in all 4 active treatment groups, and included significant increases in optically empty spaces between muscle bundles in the anterior ciliary muscle (Fig. 5), as well as an increase in myelinated nerve fiber bundles. In contrast to the study from the 1980s that used short-term PGA treatment, there was not simply a loss of ECM but, rather, an organized remodeling was seen, in which the increased spaces among ciliary muscle bundles were incompletely lined with endothelial-like cells and presumably represented new uveoscleral outflow routes (Fig. 5). There was also remodeling in the TM, including disruption of the endothelial cell monolayer of Schlemm's canal, expansion of the juxtacanalicular region of the TM, loss of ECM, and, in some samples, widening of intertrabecular spaces in the corneoscleral region of the TM,^76^ all of which could potentially contribute to an increase in outflow facility.

**FIG. 6.**
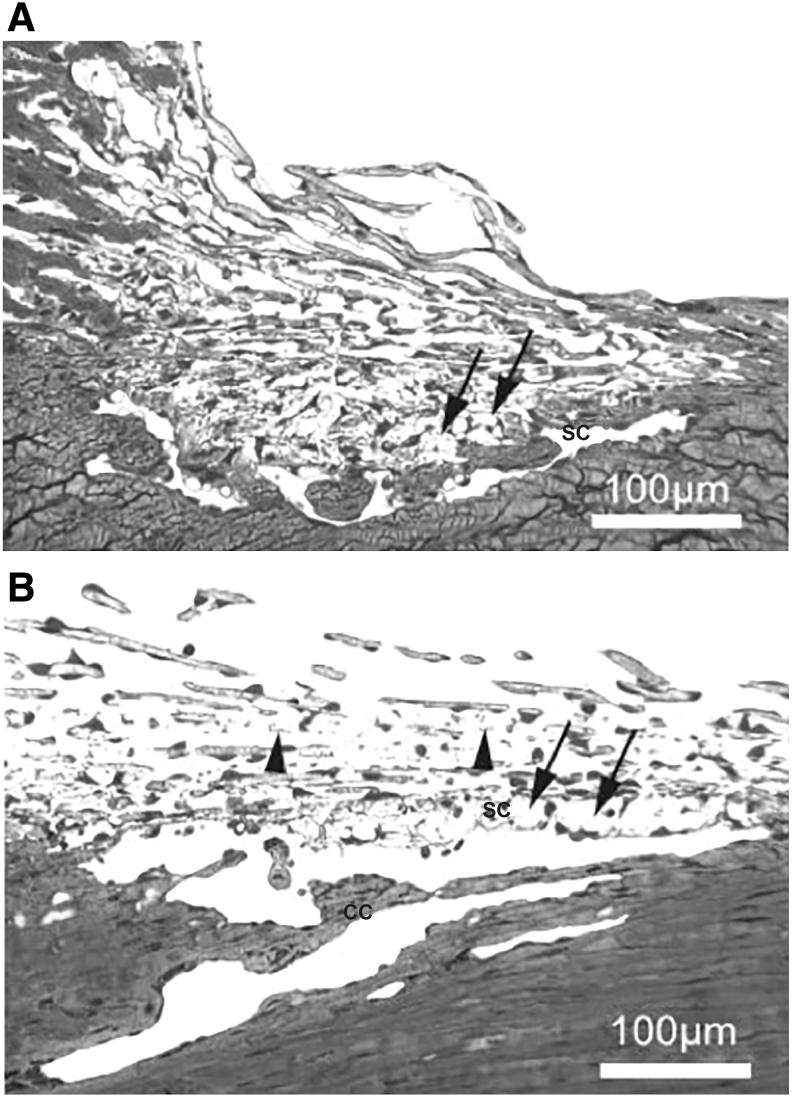
Sagittal sections through the TM of cynomolgus monkeys after 1 year of topical treatment with butaprost. Remodeling was evident in **(A)** and more pronounced in **(B)**. The juxtacanalicular region was widened and showed focal disconnections from the inner wall endothelium of Schlemm's canal (*arrows*); several lamellae were reduced in thickness or nearly eliminated (*arrowheads*). Used with permission of ARVO, from Nilsson et al.^77^; permission conveyed through Copyright Clearance Center, Inc., CC, collector channel; SC, Schlemm's canal.

Another study in cynomolgus monkeys similarly showed remodeling of the ciliary body, with apparent formation of new outflow channels, after 1 year of topical treatment with the prostaglandin EP2 agonist butaprost.^77^ TM remodeling (Fig. 6), including expansion of the juxtacanalicular region, enlargement of the collector channels and their connections to Schlemm's canal, disconnection of some TM cells from the endothelial lining of Schlemm's canal and the underlying ECM, and reduction in some TM lamellae, was evident in many of the animals.^77^ MMP expression was not evaluated in either of the primate studies, but a transient or sustained increase in MMP activity could account for the remodeling seen in the primate outflow tissues after long-term topical PGA treatment.

A recent pharmacogenetic study has provided further support for the involvement of MMPs in the mechanism of IOP lowering with PGAs.^78^ In a case–control study of 117 Spanish patients with open-angle glaucoma, candidate genes (MMP genes and the PGF2α receptor gene, *PTGFR*) and single-nucleotide polymorphisms (SNPs) of the genes were investigated for a potential relationship to responsiveness to latanoprost treatment. Although only SNPs of *PTGFR* were identified as being associated with positive or negative responses to latanoprost, subhaplotype analysis identified 6 subhaplotypes of the *MMP-1* gene associated with no response to latanoprost.^78^ These findings strongly implicate MMP-1 in the mechanism of IOP lowering with PGAs.

## Activation of MMPs by Topical PGAs in Nontarget Ocular Tissues

Activation of MMPs in the TM and the ciliary body target tissues appears to be an essential component of the mechanism of IOP lowering for the topical PGAs. However, PGA eye drops also upregulate MMPs in ocular tissues unrelated to IOP lowering. In a study using rabbits, conjunctival subepithelial collagen was increased in timolol-treated eyes compared with latanoprost-treated and control eyes, and immunostaining revealed the presence of conjunctival MMP-3 only in the latanoprost-treated eyes.^79^ Similarly, in a study using conjunctival specimens from human eyes treated with topical latanoprost or timolol, immunostaining showed greatly increased expression of MMP-1 and MMP-3 and moderately increased expression of TIMP-2 and TIMP-3 in epithelial cells and subepithelial stromal cells of latanoprost-treated eyes, and this was accompanied by a marked reduction in the stromal collagen density in the latanoprost-treated eyes relative to the timolol-treated eyes.^80^ The results of both studies suggest that PGA-induced increased MMP activity in the conjunctiva might help prevent treatment-related changes in the conjunctival ECM that contribute to failure of filtering blebs.^79,80^

The expression of MMPs in the cornea has been studied extensively as it relates to corneal wound healing.^1^ Because the stroma of the cornea is largely composed of collagen, MMP activation in the cornea might be expected to cause stromal ECM remodeling and corneal thinning. The effects of topical IOP-lowering medications, including the PGAs, on central corneal thickness (CCT) have been well studied because of the known effect of CCT on IOP measurements, that is, IOP measured with Goldmann applanation is underestimated in eyes with thinner CCT and overestimated in eyes with thicker CCT.^81^ A meta-analysis of CCT data from eyes with chronic disease has suggested that a change in CCT of 20 μm is associated with a change of 1 mm Hg in measured IOP.^24^ Topical treatment with PGAs has been shown to produce small but consistent reductions in CCT^82,83^: a reduction in mean CCT from baseline of 11 and 16 μm has been reported in glaucoma patients after 2 years of treatment with latanoprost and bimatoprost, respectively.^82^

The reduction in CCT following topical PGA treatment has been attributed to upregulation of MMPs in the corneal stroma and MMP-mediated tissue remodeling.^82^ This concept is supported by a study using cultured porcine corneal stroma cells, in which 90-min exposure to latanoprost led to changes in cell shape and an ∼50% decrease in intact fibronectin (an ECM component and substrate of MMPs, particularly MMP-13 and -14) in the cultures.^84^

The reduction in CCT associated with topical PGA treatment has no deleterious effects, but it may be of particular interest, because CCT reduction could potentially be a surrogate marker for increased MMP activity in outflow tissues. Moreover, the IOP in response to topical PGAs may be underestimated with a thinner cornea.

## Profiles of MMP Expression Vary by PGA

Clinical trials have shown some differences in efficacy among the topical PGAs, and in a network meta-analysis of randomized controlled trials that compared a single topical IOP-lowering medication with another medication or no treatment/vehicle, the relative efficacy of the topical PGAs was bimatoprost > latanoprost ≈ travoprost > tafluprost >> unoprostone.^85^ Differences in the MMP activation profiles of the individual PGAs may contribute to their differing efficacy.

In a study testing the effects of the free acids of bimatoprost, latanoprost, and unoprostone on the expression of MMPs and TIMPs in cultured human ciliary muscle cells, all of the PGAs increased the expression of MMP-1, -3, and -9, as well as TIMP-3, but only unoprostone acid decreased MMP-2 expression and increased the levels of TIMP-1 and -4. The differing effects of the PGAs on the MMP/TIMP balance reflect the relative effectiveness of the PGAs in lowering IOP, with unoprostone associated with both the lowest ratio of MMPs to TIMPs and the lowest efficacy of the PGAs.^86^ In a second study testing the effects of the free acids of bimatoprost, latanoprost, and tafluprost in human nonpigmented ciliary epithelial cell cultures, all of the PGAs increased mRNA levels for MMPs, but at equivalent concentrations, bimatoprost acid produced a greater increase in MMP-2 and MMP-3 mRNA levels compared with latanoprost acid and tafluprost acid.^87^ The results of both studies are consistent with a premise that greater efficacy of a PGA could result from greater upregulation of MMP expression and activity in ECM turnover.

## PGA Effects on MMPs Are Concentration Dependent

There is ample evidence that the upregulation of MMPs by PGAs is dependent not only on the identity of the PGA but also on its concentration. In an early study using human explant scleral cultures, the effects of latanoprost acid on MMP-3, MMP-10, MMP-14, TIMP-1, TIMP-2, and TIMP-3 mRNA expression were shown to be concentration dependent.^88^ Similarly, exposure to latanoprost acid affected the expression of mRNA for various MMPs and TIMPs in a study using cultured ciliary muscle cells from human donors.^68^ In the latter study, the direction and magnitude of effect were influenced by the cell source (donor), the particular MMP or TIMP, and the drug concentration at pharmacological and higher concentrations.^68^

**FIG. 7.**
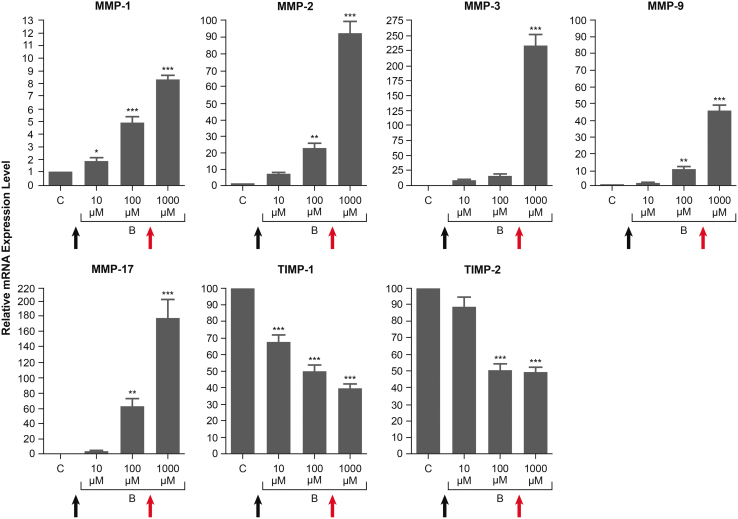
Dose-dependent effects of bimatoprost acid on levels of mRNA for MMPs and TIMPs in human nonpigmented ciliary epithelial cells. Cultures were exposed to 10, 100, or 1,000 μM bimatoprost acid (B) for 24 h or to no drug (control, C), and mRNA levels were quantified by quantitative polymerase chain reaction. *P < 0.05, **P < 0.01, ***P < 0.001 vs. control. Adapted from Yamada et al.^87^ under the terms of the Creative Commons Attribution 4.0 International License (http://creativecommons.org/licenses/by/4.0/). The superimposed *black* and *red arrows* show for comparison the maximal iris-ciliary body bimatoprost plus bimatoprost acid concentrations obtained after topical bimatoprost treatment and Bimatoprost SR administration, respectively, in a drug distribution study in beagle dogs.^102^ Bimatoprost SR, bimatoprost sustained-release implant; TIMP, tissue inhibitor of metalloproteinase.

In a more recent study using human cultured nonpigmented ciliary epithelial cells, levels of mRNA for MMPs and TIMPs were measured after the cultures were exposed to the free acid of latanoprost, bimatoprost, or tafluprost at concentrations from 10 μM to 1 mM.^87^ All 3 PGAs caused a dose-dependent increase in mRNA levels for each of the MMPs evaluated (MMP-1, -2, -3, -9, and -17) and a dose-dependent decrease in mRNA levels for TIMP-1 and TIMP-2.^87^ For bimatoprost acid, the highest tested concentration produced MMP mRNA levels up to 45-fold higher than those with the lowest tested concentration (Fig. 7).

These findings suggested to us that a high PGA concentration in outflow tissues might be able to affect the MMP/TIMP balance in a manner that produces greater MMP activity and a more sustained IOP lowering.

## Exploiting MMPs in Glaucoma Therapy: Development of a Bimatoprost Implant Providing Sustained IOP Lowering

A well understood limitation in the use of topical PGAs, and indeed all topical medications, for treatment in glaucoma is poor adherence of patients to eye drop regimens. Studies using pharmacy claims data, patient self-reports of adherence, and electronic dose-monitoring devices have reported that 14% to 90% of patients do not use their drops as prescribed.^89–94^ In line with this, although the topical PGAs are once-daily medications, a pharmacy claims-based study showed that on average, patients filled their PGA prescription and had medication available for dosing only 37% of the days in a year.^90^ The problem is compounded by the difficulty many patients have in instilling their eye drops,^95^ and the most commonly reported reasons for patients not using their eye drops as prescribed include forgetfulness, inconvenience because of the dosing frequency, lack of understanding of the disease, medication cost, and difficulty in eye drop administration.^91,93,95,96^ Patient nonadherence to glaucoma medications is a significant clinical concern, because evidence suggests that adherence to the prescribed medication is needed for optimal visual outcomes.^92,97^

**FIG. 8.**
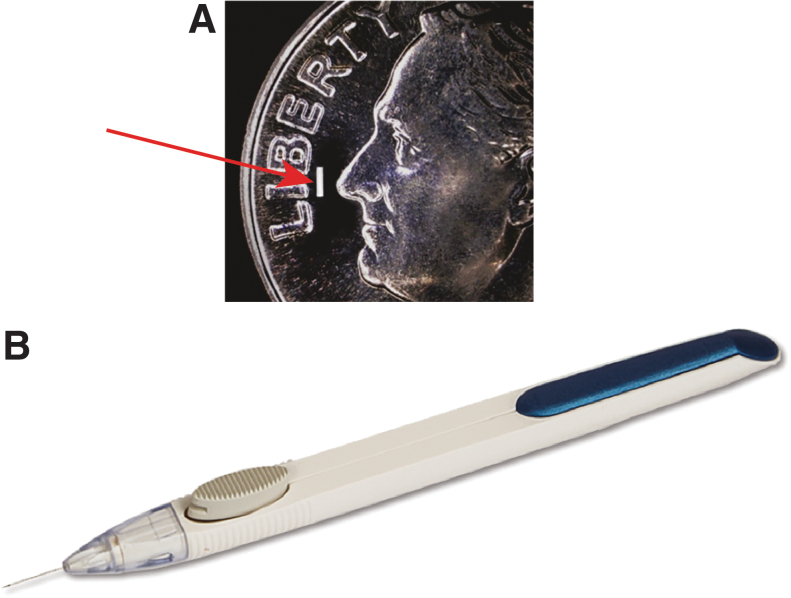
Bimatoprost SR is a first-in-class, sustained-release, bimatoprost implant that is administered intracamerally. **(A)** Photograph of an implant (10-μg dose strength; *arrow*) superimposed on an image of a dime. The implant is similar in size to the “i” in Liberty on the dime; the shape is cylindrical (radius ∼200 μm, length ∼1.1 mm). **(B)** Photograph of a single-use, 28-gauge applicator used for implant administration.

A biodegradable sustained-release bimatoprost implant (Bimatoprost SR) has been developed to address the problem of nonadherence in glaucoma and lower IOP without need for daily eye drops. Bimatoprost SR was manufactured on the Novadur (Allergan plc, Dublin, Ireland) platform for drug delivery, which uses biodegradable polymers similar to those in biodegradable sutures and has demonstrated a long track record of safety with intraocular use.^98^ The implant consists of bimatoprost embedded in a polymer matrix formulated to provide nonpulsatile, consistent drug release over time (ie, zero-order kinetics). The implant was designed to be administered intracamerally and to slowly release bimatoprost to lower IOP for 4–6 months. A single-use, 28-gauge proprietary applicator (Fig. 8) is used to place the implant into the anterior chamber of the eye in an office procedure. After administration, bimatoprost is slowly released as the matrix is hydrolyzed and metabolized to carbon dioxide and water.

The concentration of bimatoprost achieved in outflow tissues was an important consideration in the formulation of an implant with potential to provide sustained IOP lowering. It is well understood that the effects of topical PGA treatment on IOP last for several weeks after stopping use of PGA eye drops. In fact, a washout period of 4 weeks for PGAs has typically been used in clinical trials in glaucoma, and there is evidence of lingering effects of the PGAs in some patients beyond 4 weeks.^99^ We hypothesize that the continued IOP-lowering effect seen in some patients for several weeks after topical PGA washout reflects the time required for reversal of the PGA-induced MMP-mediated remodeling of outflow tissues. Because the effects of PGAs on MMP expression are concentration dependent, we reasoned that it might be possible to achieve more extensive tissue remodeling, and a longer remnant effect on IOP, if a higher drug concentration was achieved in the outflow tissues. The drug content of the 10-μg Bimatoprost SR that was developed is similar to that of a single drop of topical bimatoprost 0.03% ophthalmic solution,^100^ but the implant can achieve higher bimatoprost concentrations in outflow tissues because it takes advantage of an intracameral route for drug delivery. Thus, >80% of drug is delivered to outflow tissues, whereas <5% of drug reaches intraocular tissues after topical administration of an eye drop.^101,102^

**FIG. 9.**
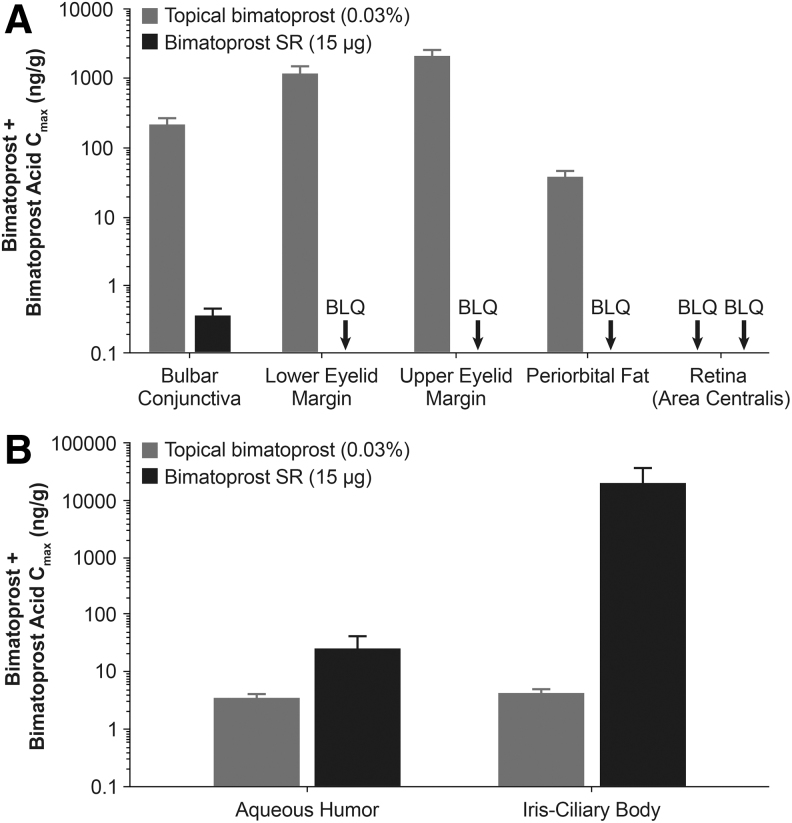
Mean maximal drug concentrations for bimatoprost plus bimatoprost acid in ocular tissues associated with PGA-related adverse effects **(A)** and associated with IOP lowering **(B)** after administration of either Bimatoprost SR 15 μg or topical bimatoprost 0.03% once daily for 7 days in beagle dogs. Republished from Seal et al.,^102^ under the terms of the Creative Commons License (http://creativecommons.org/licenses/by/4.0). *n* = 2 animals/4 eyes per time point. BLQ, below the limit of quantitation; C_max_, maximal observed concentration; PGA, prostaglandin analog/prostamide.

The ability of Bimatoprost SR to provide targeted delivery of high bimatoprost concentrations to ocular outflow tissues was demonstrated in a drug distribution study using normotensive beagle dogs.^102^ The time course of ocular drug concentrations after implant administration was consistent with the kinetics of drug release from the implant *in vitro*, where drug release is complete in less than 3 months: in the dog study, the implants had released 99.8% of their drug load and ocular tissue drug levels had significantly declined by 80 days after intracameral administration.^102^ Drug levels in ocular surface and periocular tissues such as the conjunctiva, eyelids, and periorbital fat were markedly reduced or undetectable after Bimatoprost SR 15 μg administration compared with 7 days of topical dosing with bimatoprost 0.03% (Fig. 9), suggesting the potential for reduced PGA side effects, such as conjunctival hyperemia and periorbitopathy, with the implant. In contrast, and importantly, the iris-ciliary body maximal drug concentration, measured 52 days postdose, was 4,400-fold higher after Bimatoprost SR 15 μg administration than after 7 days of topical bimatoprost 0.03% administration, and drug levels in the aqueous humor, a proxy target because of its close proximity to the TM and ciliary body, were ∼10-fold higher after Bimatoprost SR administration compared with topical dosing (Fig. 9). The high drug concentrations achieved in outflow tissues with the implant were not associated with any drug-related toxicities.^102^

It is of interest that twice-daily topical dosing of either latanoprost^103^ or bimatoprost^104^ results in decreased IOP-lowering efficacy compared with once-daily dosing. In contrast, nonclinical^105,106^ and clinical^100^ studies with Bimatoprost SR have not shown diminished IOP-lowering effects with higher drug release rates from the implant. A possible explanation is that the intraocular drug levels achieved with intracameral delivery,^102^ and the subsequent MMP upregulation in target tissues, far exceed that achieved with topical dosing, and this may overcome the ceiling effect on IOP reduction that is observed with topical dosing. Alternatively, other mechanisms of IOP lowering may be unlocked with intracameral delivery, such as reduction of the episcleral venous pressure, which has been observed with a PGA only with intracameral dosing^107^ and not with topical dosing^108^ in nonclinical studies. Sustained delivery of bimatoprost to conventional outflow tissues could potentially alter the proportion of high to low flow regions because of increased MMP activation,^109,110^ thus increasing total outflow facility.

**FIG. 10.**
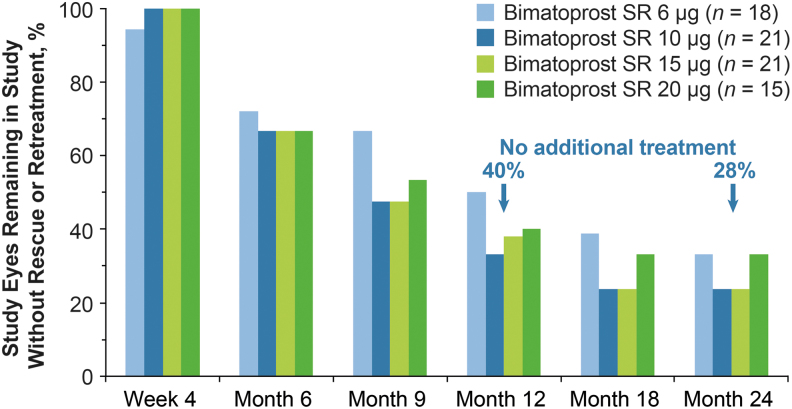
Percentage of study eyes that remained in the study with no additional IOP-lowering treatment (topical medication or laser/surgery) after a single administration of Bimatoprost SR in the phase 1/2 APOLLO study. Adapted from Craven et al.,^111^ under the terms of the Creative Commons Attribution-NonCommercial 4.0 International License (http://creativecommons.org/licenses/by-nc/4.0/).

**FIG. 11.**
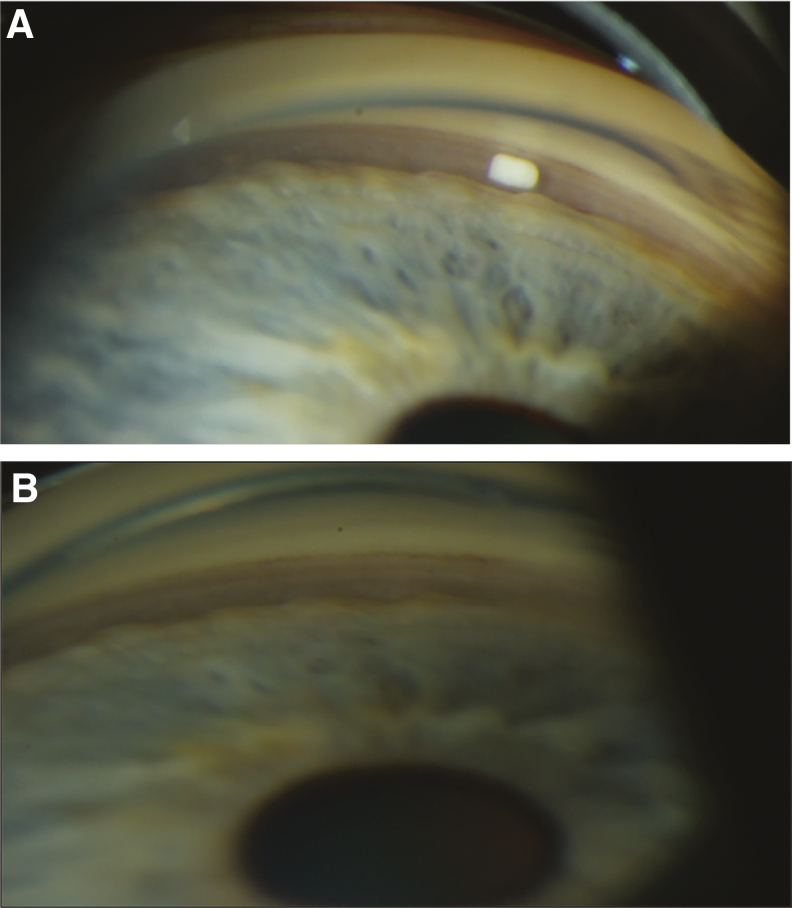
Gonioscopic photographs and IOP in a representative patient with open-angle glaucoma who demonstrated sustained IOP lowering after a single administration of Bimatoprost SR 6 μg on Day 1 in the APOLLO study. The baseline IOP was 24.5 mm Hg. **(A)** At Week 2, the implant was visible in the iridocorneal angle. **(B)** At Month 24, no residual implant was visible on gonioscopy, yet the IOP remained well controlled at 15.5 mm Hg. Reproduced by permission of E. Randy Craven, Biodegradation of Intracameral Bimatoprost Sustained-Release Implant (Bimatoprost SR) in a 24-Month, Phase 1/2 Study in Glaucoma Patients. Presented at the American Society of Cataract and Refractive Surgery 2019 Annual Meeting, May 3–7, 2019, San Diego, CA.

During the first clinical trial of Bimatoprost SR, the phase 1/2 APOLLO study,^100,111^ the formulation was modified to optimize the rate of biodegradation and drug release, and 6-, 10-, 15-, and 20-μg dose strengths of a second-generation implant were tested in 75 patients with open-angle glaucoma in the study eye. In APOLLO, patients received 1 or at most 2 administrations of implant and were followed for 2 years. The implant provided dose-dependent IOP lowering over the first 16 weeks, with IOP-lowering efficacy similar to that of a topical PGA.^100^ Remarkably, 28% of the Bimatoprost SR-treated eyes were controlled without use of any additional treatment (rescue topical medication or implant retreatment) up to 24 months after a single implant administration^111^ (Fig. 10). Because the implants contain a relatively small amount of drug (low μg), and results from nonclinical pharmacokinetics studies show nondetectable intraocular drug levels at 4.5 months after administration (Allergan, data on file, 2019), this sustained IOP lowering is unlikely to be accounted for by continued drug presence. Furthermore, in preclinical toxicology studies, there were no signs of uveitis or histologic evidence of inflammation that could be associated with a reduction of IOP (Allergan, data on file, 2015). Although residual implant depleted of drug may remain in the iridocorneal angle for weeks or months before being completely biodegraded, some of the patients who had IOP controlled up to 24 months after a single implant had no residual implant visible on gonioscopy at month 24 (Fig. 11).

The 10- and 15-μg dose strengths of Bimatoprost SR were advanced in development into phase 3 evaluation, and 2 double-masked, prospective, 20-month phase 3 studies with identical protocols (ARTEMIS 1 and 2; NCT02247804 and NCT02250651) were designed to evaluate the efficacy and safety of 3 administrations of these dose strengths of implant at 4-month intervals compared with twice-daily topical timolol 0.5% in a total of 1,122 patients with open-angle glaucoma or ocular hypertension. The ARTEMIS 1 study was recently completed; the ARTEMIS 2 study is ongoing. The primary database lock for each study occurred when the last enrolled patient completed the Week 12 visit, and all available data from the pooled dataset were analyzed. Both dose strengths of Bimatoprost SR met the primary end point of noninferiority to topical timolol in IOP lowering through Week 12 (Craven, et al. American Academy of Ophthalmology 2019 Annual Meeting, October 12–15, 2019, San Francisco, CA). Furthermore, after receiving 3 implants over 8 months, many patients had sustained IOP lowering for the remainder of the 20-month study. In the analysis of the pooled study data from the primary database lock, patients treated with Bimatoprost SR 10 μg had an 80% estimated probability of not using any additional IOP-lowering treatment (topical medication or laser/surgical procedure) for 1 year after the third administration.

These phase 3 study results showing long-term, sustained IOP lowering with Bimatoprost SR are consistent with the phase 1/2 study results, in which the estimated probability of receiving no additional treatment for 2 years after a single Bimatoprost SR 10 or 15 μg administration was 36%. We propose that the most reasonable explanation for these findings is that the high bimatoprost concentrations in target tissues after Bimatoprost SR administration alter the MMP/TIMP balance in a manner that favors more durable tissue remodeling of outflow pathways, resulting in sustained IOP lowering in the absence of continued drug exposure.

**FIG. 12.**
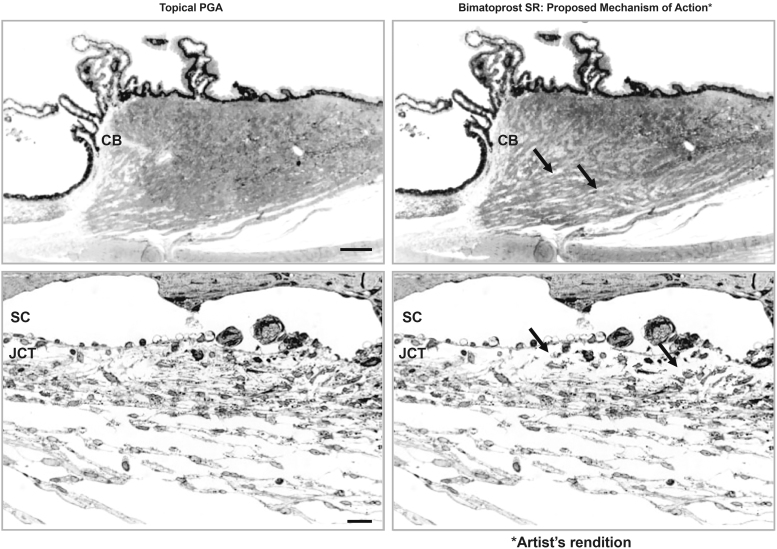
Proposed mechanism for the extended duration of effect of the bimatoprost implant. (*Left*) The mechanism of IOP lowering with topical PGAs involves remodeling of outflow pathways, seen here in the monkey ciliary body after 1 year of topical bimatoprost treatment (*top*) and in the monkey TM after 1 year of topical latanoprost treatment (*bottom*). (*Right*) Artist's rendition of the hypothesized mechanism for sustained IOP lowering with the implant. The high tissue concentrations of bimatoprost after implant administration are proposed to result in higher expression of MMPs and a shift in the MMP/TIMP balance favoring reduction in the ECM and more durable tissue remodeling, resulting in a sustained increase in aqueous outflow and a longer duration of IOP reduction. Images on *left* are used with permission of ARVO, from Morphological Changes in the Anterior Eye Segment after Long-Term Treatment with Different Receptor Selective Prostaglandin Agonists and a Prostamide, Richter et al.^76^; permission conveyed through Copyright Clearance Center, Inc., *Arrows* indicate increased areas with empty space because of ECM degradation. Scale bar: (*top*) 50 μm; (*bottom*) 20 μm. CM, ciliary body; JCT, juxtacanalicular tissue.

We hypothesize that the high drug levels produced in target tissues by the implant result in enhanced upregulation of MMPs, leading to more extensive loss of ECM in both the ciliary body and TM, as illustrated in Fig. 12. The time it takes to reverse these changes, and for the tissues to normalize, equates to the duration of IOP reduction that occurs beyond drug presence. Additional laboratory work is in progress to evaluate the differential effects of various concentrations of PGAs on the histology of the anterior segment to test this hypothesis.

The long-term clinical effects of this disease-modifying approach for treating glaucoma are currently being investigated in a 2-year extension study of the phase 3 clinical trials (ClinicalTrials.gov Identifier: NCT03891446). In the extension study, patients completing the ARTEMIS 1 and 2 studies or 2 other phase 3 studies comparing Bimatoprost SR to selective laser trabeculoplasty (NCT02250651, NCT02636946), who have not received any additional IOP-lowering medication at the original study completion, will continue follow-up to evaluate the duration of IOP lowering without requiring additional treatment.

## Conclusion

MMPs are ubiquitous in the body and have essential roles in both normal physiology and pathology. In the eye, both MMPs and the MMP/TIMP ratio in ocular tissues involved in aqueous humor outflow have an important role in the regulation of IOP. Treatment of glaucoma patients with topical PGAs increases MMP activity and ECM turnover in aqueous outflow tissues, resulting in enhanced aqueous outflow through conventional and unconventional routes and reduced IOP. By exposing tissues to high concentrations of PGA with an intracameral implant, the mechanism of IOP lowering through MMP activation can be exploited to cause durable IOP reduction, presumably because the high drug concentrations produce greater tissue remodeling compared with the lower drug levels achieved with eye drops. The magnitude of the remodeling may be influenced by factors including the dose, the PGA, and the duration of drug exposure.
